# Targeted Mass Spectrometry of a Clinically Relevant PSA Variant from Post‐DRE Urines for Quantitation and Genotype Determination

**DOI:** 10.1002/prca.202000012

**Published:** 2020-07-09

**Authors:** Joseph J. Otto, Vanessa L. Correll, Hampus A. Engstroem, Naomi L. Hitefield, Brian P. Main, Brenna Albracht, Teresa Johnson‐Pais, Li Fang Yang, Michael Liss, Paul C. Boutros, Thomas Kislinger, Robin J. Leach, Oliver J. Semmes, Julius O. Nyalwidhe

**Affiliations:** ^1^ Leroy T. Canoles Jr. Cancer Research Center Eastern Virginia Medical School Norfolk VA 23507 USA; ^2^ Department of Urology The University of Texas Health San Antonio San Antonio TX 78229 USA; ^3^ Department of Microbiology and Molecular Cell Biology Eastern Virginia Medical School Norfolk VA 23507 USA; ^4^ Mays Cancer Center at UT Health San Antonio/MD Anderson San Antonio TX 78229 USA; ^5^ Departments of Human Genetics and Urology Jonsson Comprehensive Cancer Center Institute for Precision Health University of California Los Angeles Los Angeles CA 90095 USA; ^6^ University of Toronto Department of Medical Biophysics Toronto ON M5G 1L7 Canada; ^7^ Department of Cell Systems and Anatomy The University of Texas Health San Antonio San Antonio TX 78229 USA

**Keywords:** genotype, mass spectrometry, parallel reaction monitoring, post‐digital rectal exam urine, prostate cancer

## Abstract

**Purpose:**

The rs17632542 single nucleotide polymorphism (SNP) results in lower serum prostate specific antigen (PSA) levels which may further mitigate against its clinical utility as a prostate cancer biomarker. Post‐digital rectal exam (post‐DRE) urine is a minimally invasive fluid that is currently utilized in prostate cancer diagnosis. To detect and quantitate the variant protein in urine.

**Experimental design:**

Fifty‐three post‐DRE urines from rs17632542 genotyped individuals processed and analyzed by liquid chromatography/mass spectrometry (LC‐MS) in a double‐blinded randomized study. The ability to distinguish between homozygous wild‐type, heterozygous, or homozygous variant is examined before unblinding.

**Results:**

Stable‐isotope labeled peptides are used in the detection and quantitation of three peptides of interest in each sample using parallel reaction monitoring (PRM). Using these data, groupings are predicted using hierarchical clustering in R. Accuracy of the predictions show 100% concordance across the 53 samples, including individuals homozygous and heterozygous for the SNP.

**Conclusions and clinical relevance:**

The study demonstrates that MS based peptide variant quantitation in urine could be useful in determining patient genotype expression. This assay provides a tool to evaluate the utility of PSA variant (rs17632542) in parallel with current and forthcoming urine biomarker panels.

## 1 Introduction

It is estimated that more than 31 000 males died from prostate cancer (PCa) in 2019 making it the second leading cause of cancer‐related death among US males.^[^ ^1^ ^]^ As is the case with other malignancies, early detection and treatment of prostate cancer provide the greatest chance for more favorable patient outcomes. Prostate specific antigen(PSA) is the most widely used biomarker for PCa screening, diagnosis, risk stratification, and monitoring. PSA was first discovered in the 1970s and first measured in blood in 1980 by Papsidero et al.^[^ ^2^, ^3^ ^]^ It was not until a large study published in 1987 from Stanford University by Stamey et al. that PSA was widely recognized as a biomarker of PCa.^[^ ^4^ ^]^ PSA has since become the most commonly used biomarker in all of oncology, however, several shortcomings with respect to specificity and sensitivity of the assay have been recognized.^[^ ^5^, ^6^ ^]^ This has resulted in cases of both under‐ and overtreatment of the disease.^[^ ^7^, ^8^, ^9^, ^10^, ^11^ ^]^

PSA testing from blood is historically reported as normal if the value is <4 ng mL^−1^. This typically results in a physician not pursuing any additional testing or examination of the prostate. The most common issue with PSA being an elevated false‐positive rate for PCa driven by non‐malignant conditions like benign prostatic hyperplasia. Nevertheless, false‐negatives also occur, albeit at a lower frequency. The Prostate Cancer Prevention Trial included 2950 men that never had a PSA level higher than 4 ng mL^−1^ over a 7 year period.^[^ ^12^, ^13^ ^]^ Each participant underwent a prostate biopsy at the end of the study and 15.2% (449 men) were subsequently diagnosed with PCa.^[^ ^12^ ^]^ All of these 449 men would have been missed using only the standard 4 ng mL^−1^ PSA threshold.

Genetic factors are among the many elements that influence an individual's PSA level, and indeed many aspects of prostate cancer biology.^[^ ^14^ ^]^ There are multiple reports of various single‐nucleotide polymorphisms (SNPs) correlating with serum PSA levels.^[^ ^15^, ^16^, ^17^, ^18^, ^19^, ^20^ ^]^ One SNP that repeatedly shows this connection is rs17632542 on chromosome 19 in the kallikrein‐3 gene leading to lower serum PSA levels than expected.^[^ ^17^, ^18^, ^19^, ^21^, ^22^, ^23^ ^]^ The genetic variant alters a codon ATT to ACT leading to an amino acid substitution of an isoleucine to a threonine at position 179 (I179T). Genotypes of TT are homozygous wild‐type, CT are heterozygous, and CC are homozygous variant. Currently there is no method to directly assess protein expression of these allelic alterations that would allow for better understanding of phenotypic variability.

**Clinical Relevance**

Prostate cancer is the second leading cause of cancer‐related death amongst men in the US. The most widely used screening technique is the measurement of levels of prostate specific antigen (PSA) in serum. A genetic variant of PSA, rs17632542, leads to lower serum PSA levels than would be expected based on other clinical prognostic features. To provide a method for the sensitive and specific detection of the variant PSA protein in post‐digital rectal exam (post‐DRE) urine samples, we developed a targeted mass spectrometry assay. This assay utilizes minimal sample volumes together with a high‐throughput processing protocol in a 96‐well format, making it highly efficient for screening. We propose multiplexing this assay with our previously identified aggressive disease markers, as well as existing clinical biomarkers such as Prostate cancer antigen 3 (PCA3), TMPRSS2:ERG (T2:ERG), etc. to assist in the management of prostate cancer especially in low‐risk cohorts. We also note that our direct protein assay is a useful means to evaluate allelic‐specific protein levels and overcome the uncertainty of phenotypic expression due to significant heterozygosity at this allele. The clinical utilization of current Food and Drud Administration (FDA) approved post‐DRE urine biomarkers for prostate cancer detection and monitoring provide an opportunity for parallel assessment of a PSA variant that may impact overall risk determination.

Urine collected after a prostate proximal post‐digital rectal exam (post‐DRE) was used as the biological material for this study. This fluid can be collected from a patient after a routine DRE and has previously been shown by our group and others to be a good source of prostate proteins including PSA, and thus an excellent biomarker substrate.^[^ ^24^, ^25^, ^26^, ^27^, ^28^ ^]^ The objective of this study was to detect and quantify the abundance of I179T PSA, and to make genotype‐specific protein expression classifications using targeted mass spectrometry (MS) data.^[^ ^29^ ^]^ This approach would allow for evaluation of PSA variant rs17632542 protein expression as a component of multiplexed assays in the management of men in low‐risk cohorts that utilize patient urine.

## 2 Results

### 2.1 Stable‐Isotope Labeled and Endogenous PSA Peptide Detection

**Figure 1:**
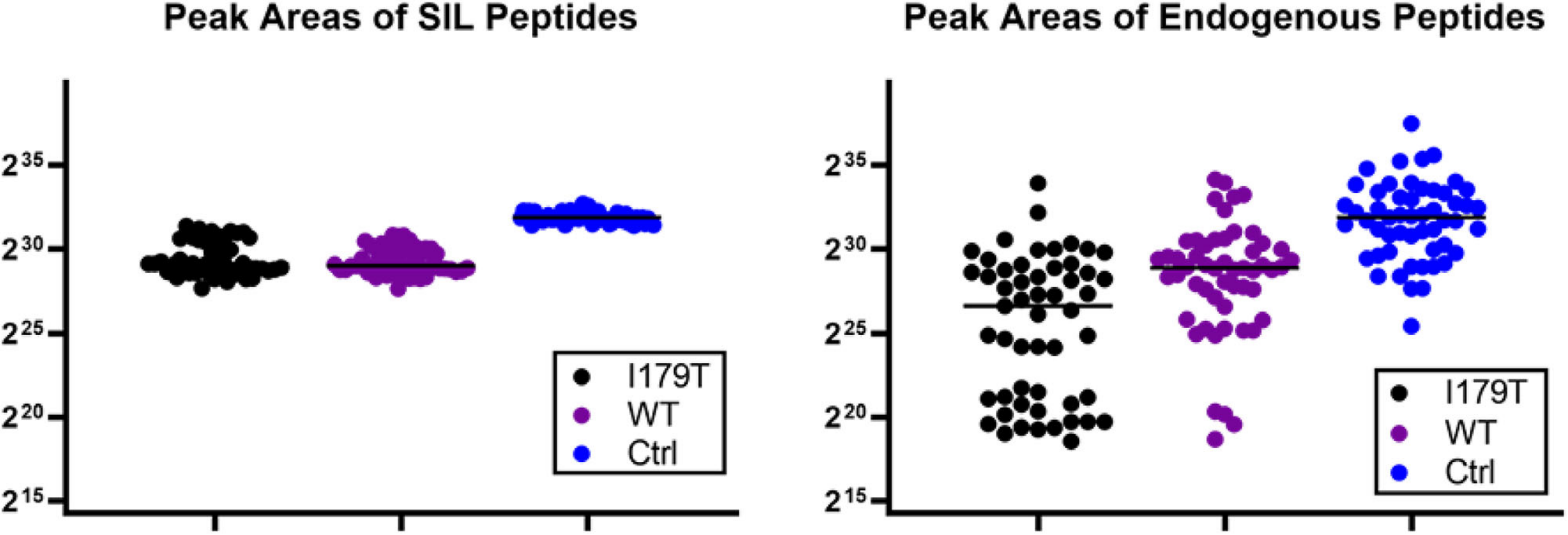
SIL and Endogenous Peptide Presence. The levels, as determined by summation of MS2 peak integrations by Skyline, of each of the stable‐isotope labeled (SIL) and endogenous peptides are plotted across each of the 53 samples. The group medians are indicated with the black line. A) The SIL peptides were present in all the samples, as expected. They were used to aid in the detection, verification, and quantitation of the corresponding endogenous peptides. The SIL control peptide was also used to back calculate absolute amounts of PSA. B) The endogenous peptides were more variable across the samples which is expected based on person‐to‐person variability.

Each of the 53 samples were spiked with three unique stable‐isotope labeled (SIL) peptides corresponding to the control peptide (LSEPAELTDAVK), the wild‐type (WT) peptide (LQCVDLHVISNDVCAQVHPQK), and the I179T variant peptide (LQCVDLHVTSNDVCAQVHPQK). The control peptide is proteotypic and unique to PSA and was used to verify the presence of PSA in each sample by comparing the SIL peptide to the endogenous. The SIL and endogenous control peptides were consistently detected in each of the 150 parallel‐reaction monitoring (PRM) analyses performed. This confirmed the presence of PSA in all the post‐DRE urine samples that were analyzed. The other two peptides, WT and I179T, were also included as targets in the multiplexed PRM assay, and their SIL versions were again detected in all the 150 acquisitions as expected. The levels of the SIL peptides are observed across all the samples in **Figure** **1**A. As expected based on the three genotypes present in the sample set, the detection of the endogenous versions of the I179T and WT peptides was variable. The levels of each of the endogenous peptides are shown in Figure 1B.

The endogenous version of I179T was detected in 99 of 150 runs, and the endogenous WT peptide in 138 of the 150. As each of the 53 samples had technical duplicates and 44 of 53 had technical triplicates, the presence or absence among the replicates was compared. Each technical replicate showed the same pattern of presence and absence for all three of the SIL and endogenous peptides.

### 2.2 Genotype Prediction by Proteomics

**Figure 2:**
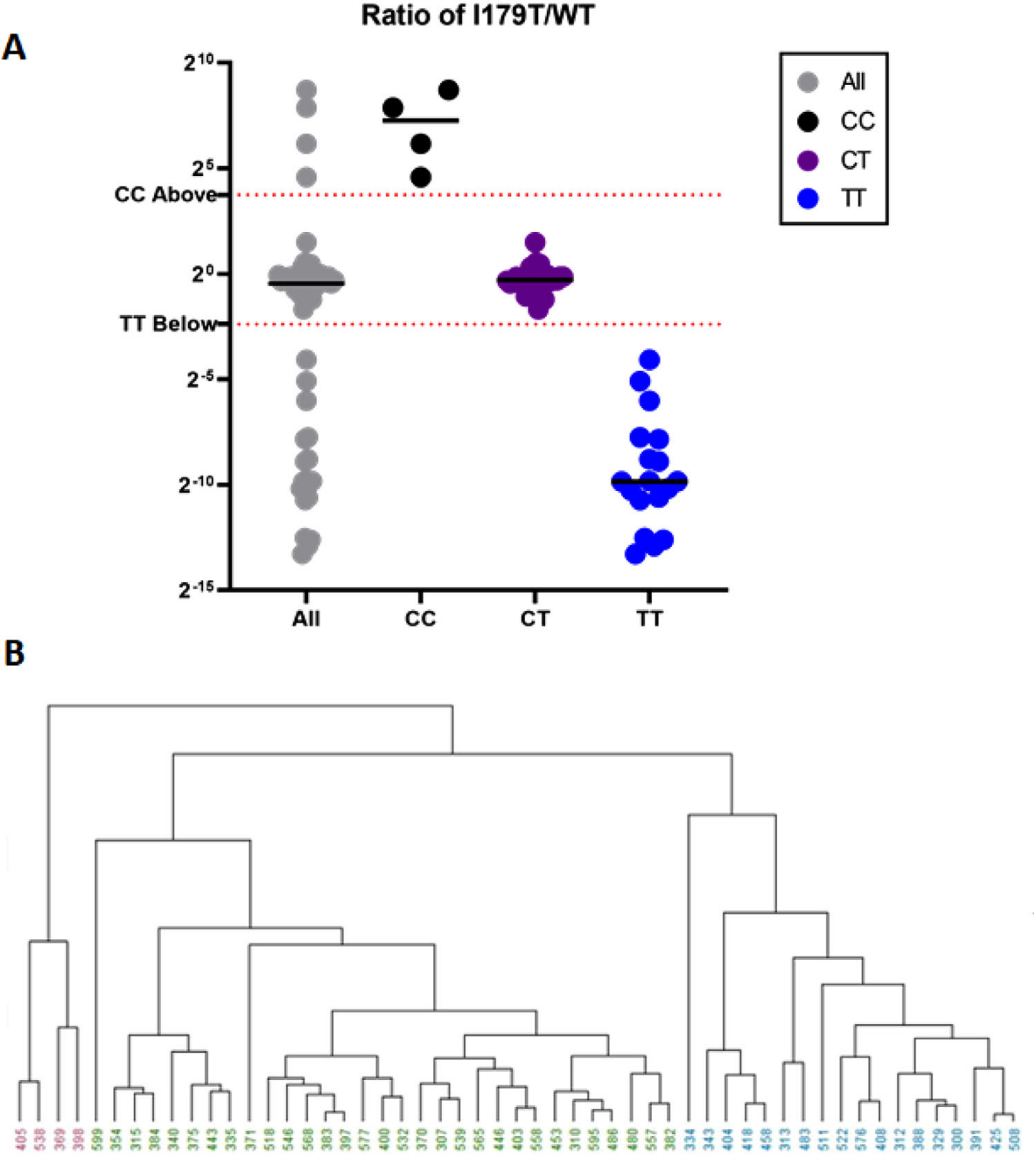
Grouping and Clustering for Genotype Prediction. A) Ratio values from I179T⁄WT were plotted for all 53 samples. Using the data here along with C initial genotype classifications were made as well as preliminary grouping thresholds. B) Hierarchical clustering shows potential groupings across all 53 samples. In this case, the information that there should be three groups was provided to the algorithm as there are three possible genotypes.

The study was performed in a double‐blinded randomized fashion and genotype predictions were made based on the acquired MS data. Integrated fragment ion peak areas from the control, WT, and I179T peptides were imported into R for initial analysis. To look for grouping patterns hierarchical clustering, **Figure** **2**B, was carried out in R.

Distance calculations for hierarchical clusters were calculated using the maximum distance between two components and clustering was performed using an unweighted pair grouping method with arithmetic means. The dendrogram was set to cut at the lowest point that would yield three clusters. The various groups are indicated by different colors as seen in Figure 2B and were set without user intervention.

A ratio value for each ${}^{I179T}{/}_{\text{WT}}$ peptide area was plotted in Figure 2A and Figure S1, Supporting Information. We determined a preliminary threshold for classification by taking the midpoint between the highest and lowest ratio value across the predicted groups. For example, the highest value for a sample with the predicted TT genotype was 0.06 and the lowest value for a CT sample was 0.31. The midpoint between these two yielded a threshold of 0.19. Values ≤0.19 were predicted to be of the homozygous wild‐type group, TT. Likewise, the midpoint between the highest CT value, 2.80, and the lowest CC value, 23.79, results in a threshold of 13.3. The final predicted groupings were thus determined using the ratio value mentioned above and the following formulas: TT≤0.19, CC≥13.3, and 0.19<CT<13.3. Panel A from Figure 2 shows how these thresholds fit within the entire dataset. Table S2, Supporting Information shows the concentrations of the spiked peptides that were used in the 53 post‐DRE urine samples. The results from non‐targeted data‐dependent acquisition (DDA) analyses were used to identify and characterize the post‐DRE urine total proteome. The results of these analyses are similar to our published post‐DRE urine proteome data (24, 31). Mascot search results demonstrate the complexity of the post‐DRE urine proteome (Figure S3, Supporting Information). This underscores the specificity of our PRM assay in detecting the PSA mutant peptides.

### 2.3 PSA Levels

**Figure 3:**
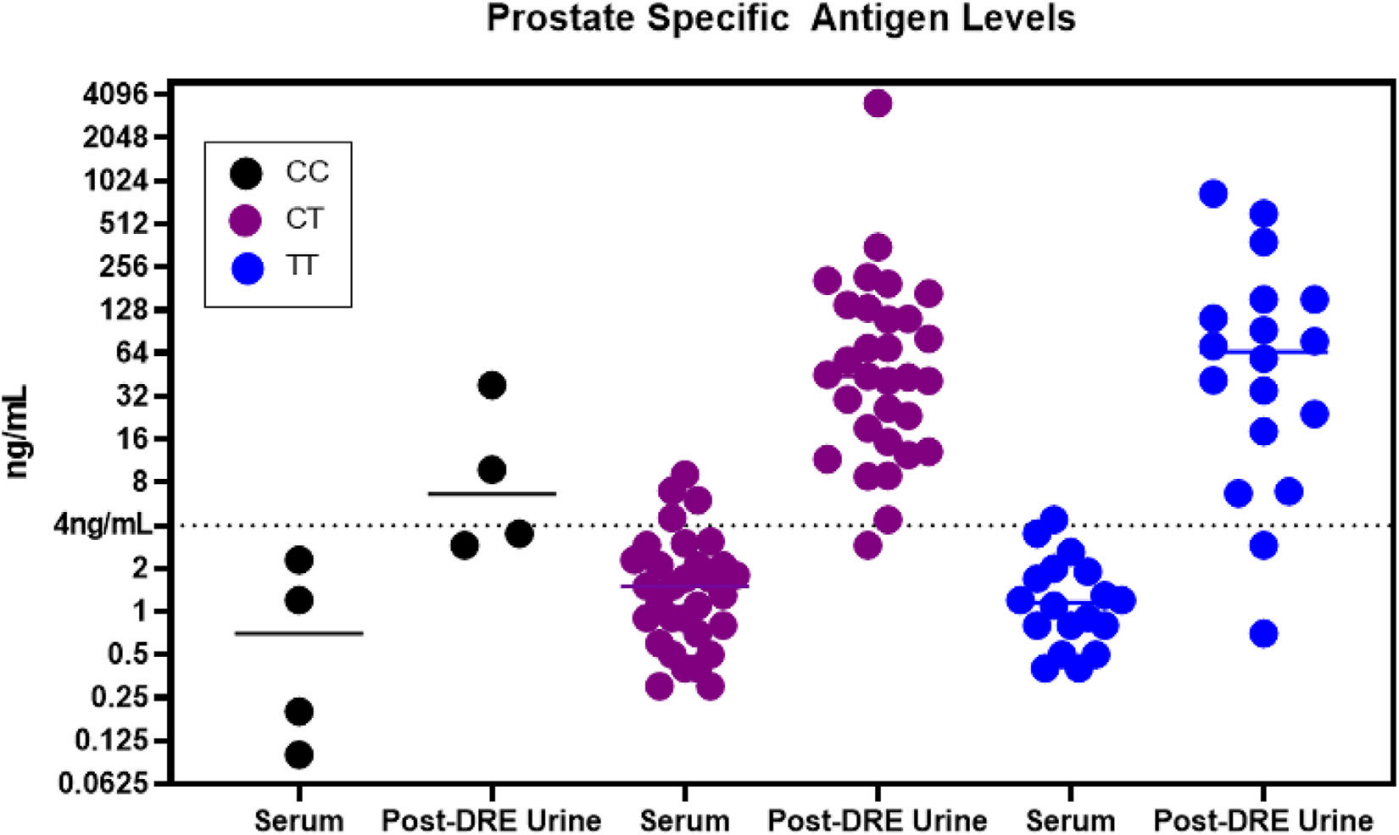
Prostate Specific Antigen Levels. Prostate specific antigen (PSA) levels are shown across the various genotypes and sample sources. PSA is consistently more concentrated in post‐DRE urines that increases the sensitivity of this assay compared to serum. In addition, the trends across the genotypes are mirrored between the two sample sources.

Serum PSA concentrations were determined using standard clinical protocols in ng mL^−1^. These values were separated by genotype (**Figure** **3**). The control SIL peptide was used to back‐calculate the concentration of PSA in each post‐DRE urine sample using MS data. The two sample sources show similar trends across the genotypes although the Spearman's correlation coefficient is not statistically significant (*p* = 0.07) (Figure S2, Supporting Information). Post‐DRE urines consistently show higher concentrations which might be expected based on the proximity to the prostate. A listing of the samples and their PSA levels in ng mL^−1^ is provided (**Table** **1**).

**Table 1:** Sample Information Table. The table shows the 53 samples along with genotype information. PSA concentrations in nanograms per milliliter (ng mL^−1^) are also displayed from both serum and post‐DRE urines. Post‐DRE urine consistently displayed higher PSA levels

Samples	Genotype	Serum PSA [ng mL^−1^]	Post‐DRE Urine PSA [ng mL^−1^]	Samples	Genotype	Serum PSA [ng mL^−1^]	Post‐DRE Urine PSA [ng mL^−1^]
300	TT	1.2	152.2	408	TT	0.5	18.1
307	CT	1.7	205.8	418	TT	1.2	382.6
310	CT	1.5	45.2	425	TT	0.8	71.4
312	TT	1.9	77.2	443	CT	2.1	4.4
313	TT	0.8	6.9	446	CT	1.1	217.9
315	CT	0.3	8.9	453	CT	2.3	43.4
329	TT	3.5	92.2	458	TT	1.1	604.4
334	TT	0.4	0.7	480	CT	1.8	69.2
335	CT	0.4	8.8	483	TT	4.4	2.9
340	CT	0.9	2.9	486	CT	3.1	110.8
343	TT	0.4	152.0	508	TT	2.6	41.3
354	CT	0.9	15.3	511	TT	0.5	6.7
369	CC	0.2	9.8	518	CT	1.7	80.7
370	CT	1.3	352.5	522	TT	0.8	58.7
371	CT	1.1	195.0	532	CT	0.5	19.1
375	CT	2.1	11.6	538	CC	0.1	3.5
382	CT	0.3	40.9	539	CT	0.5	167.3
383	CT	7.0	57.0	546	CT	2.9	26.2
384	CT	0.6	12.4	557	CT	6.0	30.3
388	TT	0.9	112.3	558	CT	9.1	131.9
391	TT	1.7	34.9	565	CT	1.5	109.7
397	CT	4.5	40.8	568	CT	0.8	44.1
398	CC	1.2	38.3	576	TT	1.3	24.0
400	CT	0.7	23.2	577	CT	0.9	13.1
403	CT	3.0	138.8	595	CT	2.1	69.3
404	TT	2.0	834.5	599	CT	0.4	3573.6
405	CC	2.3	2.9				

Overall, the average serum PSA levels across the 53 samples was 1.8 ng mL^−1^. The lowest sample had only 0.1 ng mL^−1^ and corresponded to a homozygous variant individual. The highest level was 9.1 ng mL^−1^ from a heterozygote. In the post‐DRE urines, the overall average was 96.6 ng mL^−1^ with 0.7 and 834.5 ng mL^−1^ being the minimum and maximum, respectively. The calculated amount of PSA for one of the post‐DRE urine samples was extremely high and was excluded as an outlier for the calculations listed immediately prior and does not alter any predicted groupings. The PSA values for this sample are still included in **Figure** **3** and Table 1.

## 3 Discussion

To our knowledge, our current study and data present the first MS detection and quantitation of the I179T PSA proteoform resulting from the rs17632542 SNP in post‐DRE urine. This prostate proximal fluid can be easily collected and processed using high‐throughput methods of small clinical sample volumes in large batches by our optimized MStern protocol. Each assay requires only a minimal volume of 250 μL. To further increase throughput, isobaric reporter tags, for example, tandem‐mass tag, could be incorporated allowing for a single MS acquisition to provide accurate genotype information and quantitation on upward of 11 individuals at once. Additionally, gradient lengths can be significantly shortened, and chromatographic conditions optimized for speed if needed. In fact, acquisition parameters are under optimization to allow for a more than 75% shorter run time per sample with no hardware changes (Figure S4, Supporting Information).

The accuracy of determining not only the presence of the I179T variant, but also the corresponding WT peptide allowing for genotype determination was 100%. This is an important point as studies including more than 1300 patients have shown that serum PSA levels are lower for individuals carrying two copies of the rare allele than heterozygous individuals and levels are highest among homozygous wild‐type individuals.^[^ ^19^ ^]^ The overall expectation that homozygous individuals of the rare allele result in lower PSA levels is evident from both serum PSA, and the MS measurement of the control PSA peptide as seen in Figure 3. However, the homozygous WT and heterozygous difference was not readily observed in our data, but we suspect this may present with larger sample numbers.

Although we cannot be certain of the number of PCa cases that would have been treated differently had the presence and quantitation of the rs17632542 SNP been known, a multiplexed urine‐based assay that incorporates our current approach will allow for rapid and accurate determination of genotype expression. In fact, the ability to assess allele‐specific protein expression may assist in stratifying risk associated with heterozygosity at this site. Various theories exist including half‐life reduction due to variant protein instability and overall deficiency of secretion. Sampling from post‐DRE urines may help remedy these issues as the massaging of the prostate during a DRE aids in the release of PSA amongst other prostate proteins immediately prior to sample collection.^[^ ^30^ ^]^ Figure 3 shows that broadly PSA concentrations among diseased patients are higher in this proximal fluid than in serum. Although, we do acknowledge that a normalization would likely need to be considered such as to the protein uromodulin to account for variability in sample concentrations.

## 4 Associated Data

All data for this study has been uploaded and is publicly available. Processed Skyline data can be found at: https://panoramaweb.org/Dc9U7C.url Raw data files are available at ProteomeXchange under ID PXD017257.

## 5 Experimental Section

##### Sample Preparation

Blinded and randomized post‐DRE urines were processed through an adapted MStern protocol first published by Berger et al.^[^ ^31^ ^]^ The protocol utilizes a 96‐well plate with porous polyvinylidene fluoride membranes (Sigma Millipore MSIPS4510). Briefly, three 250 µL pre‐spun aliquots of 53 post‐DRE urine samples were reduced using 5 mm dithiothreitol for 30 min at 56 °C. After cooling, samples were alkylated with 25 mm iodoacetamide to prevent disulfide bond reformation. Samples were bound to the membranes using a vacuum manifold. Digestion was carried out on the membrane at 37 °C for 4 h using 50 µL per well of a solution containing 1 µg Trypsin/Lys‐C, 100 mm ammonium bicarbonate, 5% acetonitrile, and 1 mm CaCl_2_. Peptides were collected by passing 50 µL of 50% acetonitrile through each well twice by centrifugation for 2 min at 2500 × *g*. Samples were dried using a SpeedVac. Finally, peptides were purified using solid‐phase extraction (SPE) C18 tips (Pierce 87784) according to manufacture instructions.

##### LC‐MS Analysis

SPE processed peptides were dried and suspended in 15 µL of 0.1% formic acid. Peptide concentrations were determined by NanoDrop and SIL peptides were spiked at detectable concentrations. For each injection, 2 µg of total peptide was loaded onto an in‐line EASY‐Spray 50 cm C18 column (Thermo Fisher ES803A) using an EASY‐nLC‐1200 UHPLC system. Data were acquired with both a full MS1 scan and an unscheduled PRM scan targeting both endogenous and SIL peptides of interest over a 140‐min gradient using a Thermo Fisher Orbitrap Fusion Lumos mass spectrometer. Targets were as follows: I179T variant, LQCVDLHV**T**SNDVCAQVHPQK, endogenous *m*/*z* = 612.799 SIL *m*/*z* = 614.803; WT, LQCVDLHVISNDVCAQVHPQK, endogenous *m*/*z* = 615.809 SIL *m*/*z* = 617.812; control, LSEPAELTDAVK, endogenous *m*/*z* = 636.838 SIL 640.845; additional control without SIL, HSQPWQVLVASR, *m*/*z* = 704.378. This information is summarized in Table S1, Supporting Information. All samples were run in technical duplicates and 44 of the 53 had technical triplicates resulting in 150 raw PRM files. A randomized group of 16, 30%, samples were also acquired in DDA mode using identical chromatographic conditions to characterize the proteome of post‐DRE urine samples. All samples were blinded, randomized using an online random number generator, and again randomized prior to MS acquisition.

##### Data Processing and Analysis

Peak detection and integration was performed using Skyline (v19.1.0.193). A panel of MS2 fragment ions were used for both quantitation and verification for each peptide. Unique fragment ions were included to distinguish between the I179T and WT peptide including a characteristic series of consecutive y‐ions y15++, y14+, y13+ as well as LC retention times. Mascot (Matrix Science, UK) database searches were used for further verification and validation of peptide identifications. Summed MS2 peak integrations were exported to Microsoft Excel where areas were averaged per sample. Initial visualization and grouping predictions were performed using the “stats,” “ggplot2,” and “dendextend” packages within R (v3.5.0). GraphPad Prism (v8.3.0) was used for generating scatter plots.

## Conflict of Interest

The authors declare no conflict of interest.

## Supporting information

Supporting Information

Supporting information
