# Supplementary material for: Targeted Mass Spectrometry of a Clinically Relevant PSA Variant from Post‐DRE Urines for Quantitation and Genotype Determination
Source: Proteomics Clin Appl. 2020 Jul 9;14(6):2000012. doi: 10.1002/prca.202000012 (PMC7674190; doi:10.1002/prca.202000012)
Supplement: Supplementary file 1 — Supporting Information [file PRCA-14-2000012-s001.docx]

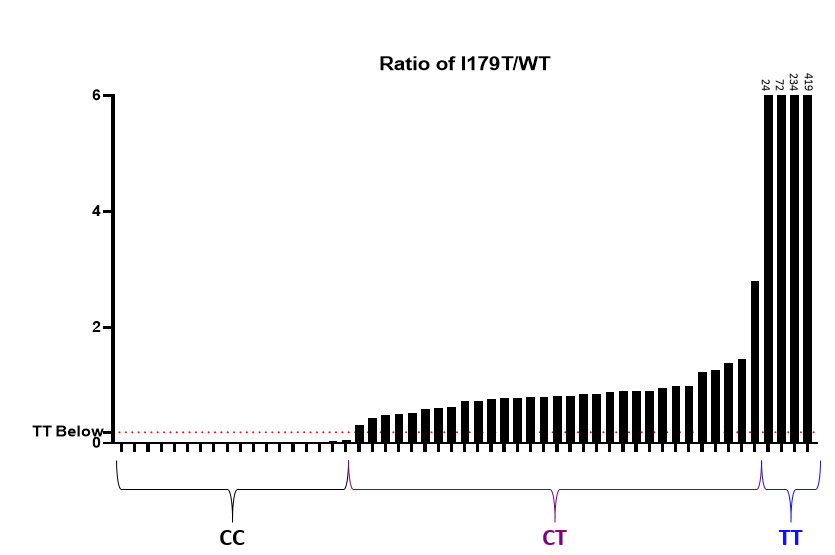


**Supplemental Figure 1: Peak Area Ratio of I179/WT Peptides.** Bar chart showing the ratio of integrated peak areas for the I179T variant peptide and WT peptide. The genotype specification of TT (homozygous variant) can be seen below the dotted line. The figure shows how the ratio of these two peptides of interest helps distinguish an individual’s genotype.


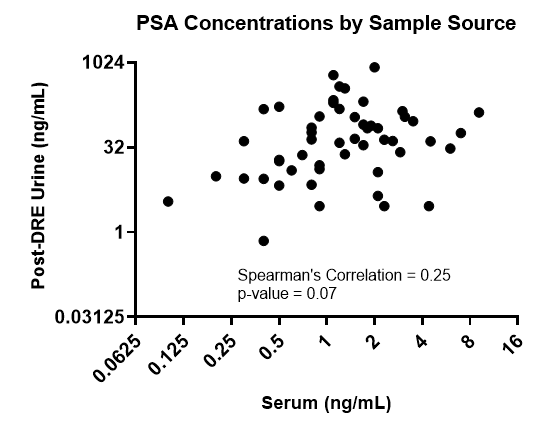


**Supplementary Figure 2: PSA Concentrations by Sample Source.** Scatter plot for investigating the correlation between the PSA levels from two sample sources across each of the individuals in this study. Although the results are not statistically significant, the plot appears to show positive correlation.

#

# Supplemental Figure 3: Mascot Protein Identifications for Representative Post-DRE Urine Samples DDA Acquisition. Shown here are protein identifications obtained by searching DDA acquired data using the Mascot search engine. The information here is a representative proteomic profile of post-DRE urine sample # 568. The overall profile matches previously published post-DRE urine data.

#
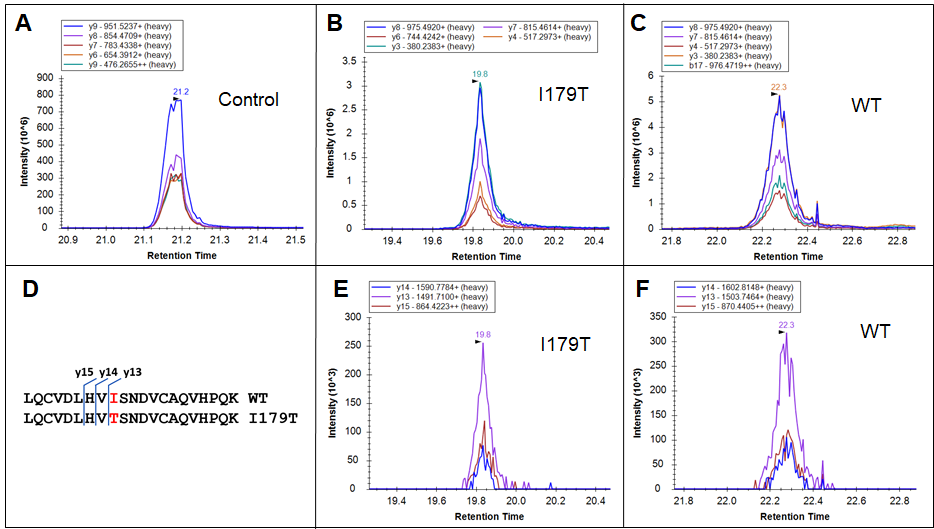
Supplemental Figure 4: Chromatograms from 30 min LC gradients showing the XICs and retention time separations for the WT, Mutant and Control Peptide 1.

The chromatograms below show several extracted ion chromatograms (XICs) for the peptides that were discussed in this paper: I179T, WT, Control Peptide 1. The XICs are from the significantly abbreviated 30 minute gradient mentioned in the manuscript. Each of the peptides was detected and showed chromatographic separation in this more than 75% reduction in gradient time. . (A-C) MS2 extracted-ion chromatograms (XICs) of the top 5 most intense fragments ions for (A) control, (B) I179T, and (C) WT SIL peptides. (D) Diagram showing a selected series of consecutive y-ions (y15++, y14+, y13+) which distinguish between I179T and WT peptides. (E-F) MS2 XICs displaying a series of consecutive distinguishing y-ions (y15++, y14+, y13+) for (E) I179T and (F) WT SIL peptides. For all XICs, retention time (minutes) of the most intense peaks are labeled. The 30 minute gradient enabled at least a 1 minute separation (retention time difference) between each peptides.

| **Identifier** | **Peptide Sequence** | **m/z** |
| --- | --- | --- |
| I179T | LQCVDLHVTSNDVCAQVHPQK | 612.7994 (+4) |
| I179T heavy | LQCVDLHVTSNDVCAQVHPQK | 614.8030 (+4) |
| WT | LQCVDLHVISNDVCAQVHPQK | 615.8085 (+4) |
| WT heavy | LQCVDLHVISNDVCAQVHPQK | 617.8121 (+4) |
| Ctrl | LSEPAELTDAVK | 636.8377 (+2) |
| Ctrl heavy | LSEPAELTDAVK | 640.8448 (+2) |
| Ctrl2 | HSQPWQVLVASR | 704.3782 (+2) |

**Supplemental Table 1: The table below shows the targets of the PRM assay**. Charge states were selected that consistently provided the most intense signal for each target.

# Supplemental Table 2: Spiked Levels of SIL Peptides

| **Sample #** | **fmol Injected of Each SIL Peptide** | **Sample #** | **fmol Injected of Each SIL Peptide** |
| --- | --- | --- | --- |
| 300 | *331 fmol* | 408 | *324* *fmol* |
| 307 | *327* *fmol* | 418 | *325* *fmol* |
| 310 | *325* *fmol* | 425 | *333* *fmol* |
| 312 | *329* *fmol* | 443 | *304* *fmol* |
| 313 | *335* *fmol* | 446 | *328* *fmol* |
| 315 | *325* *fmol* | 453 | *326* *fmol* |
| 329 | *332* *fmol* | 458 | *328* *fmol* |
| 334 | *334* *fmol* | 480 | *336* *fmol* |
| 335 | *329* *fmol* | 483 | *291* *fmol* |
| 340 | *327* *fmol* | 486 | *322* *fmol* |
| 343 | *327* *fmol* | 508 | *335* *fmol* |
| 354 | *327* *fmol* | 511 | *333* *fmol* |
| 369 | *333* *fmol* | 518 | *331* *fmol* |
| 370 | *337* *fmol* | 522 | *335* *fmol* |
| 371 | *322* *fmol* | 532 | *326* *fmol* |
| 375 | *327* *fmol* | 538 | *327* *fmol* |
| 382 | *329* *fmol* | 539 | *337* *fmol* |
| 383 | *338* *fmol* | 546 | *340* *fmol* |
| 384 | *335* *fmol* | 557 | *329* *fmol* |
| 388 | *328* *fmol* | 558 | *327* *fmol* |
| 391 | *331* *fmol* | 565 | *324* *fmol* |
| 397 | *337* *fmol* | 568 | *324* *fmol* |
| 398 | *326* *fmol* | 576 | *332* *fmol* |
| 400 | *341* *fmol* | 577 | *332* *fmol* |
| 403 | *336* *fmol* | 595 | *329* *fmol* |
| 404 | *320* *fmol* | 599 | *324* *fmol* |
| 405 | *333* *fmol* |  |  |

**Supplemental Table 2:** **Spiked Levels of SIL Peptides**. The table here shows the levels of the spiked SIL peptides (I179T, WT, and Control Peptide 1) across each of the samples. Unfortunately, due to normalizing injection amounts to maintain the blinded status of the samples, levels vary slightly across the entire sample set. All variations in SIL levels were accounted for in back-calculated concentrations discussed and listed for this manuscript.
